# Efficacy of celecoxib add-on treatment for immuno-metabolic depression: Protocol of the INFLAMED double-blind placebo-controlled randomized controlled trial

**DOI:** 10.1016/j.bbih.2022.100585

**Published:** 2022-12-30

**Authors:** J.C. Zwiep, P.M. Bet, D. Rhebergen, M.T. Nurmohamed, C.H. Vinkers, B.W.J.H. Penninx, Y. Milaneschi, F. Lamers

**Affiliations:** a Department of Psychiatry, Amsterdam UMC, Vrije Universiteit, De Boelelaan 1117, 1118, 1081 HV, Amsterdam, the Netherlands; b Amsterdam Public Health, Mental Health Program, Amsterdam, the Netherlands; c Amsterdam Neuroscience, Mood, Anxiety, Psychosis, Sleep & Stress Program, Amsterdam, the Netherlands; d Department of Clinical Pharmacology and Pharmacy, Amsterdam UMC, Vrije Universiteit, De Boelelaan 1117, 1118, 1081 HV, Amsterdam, the Netherlands; e GGZ Centraal Mental Health Care, Department of Research, Amersfoort, the Netherlands; f Department of Rheumatology, Amsterdam UMC, Vrije Universiteit, De Boelelaan 1117, 1118, 1081 HV, Amsterdam, the Netherlands; g Department of Anatomy & Neurosciences, Amsterdam UMC, Vrije Universiteit Amsterdam, De Boelelaan 1117, 1118, 1081 HV, Amsterdam, the Netherlands; h GGZ InGeest Mental Health Care, 1081 HJ, Amsterdam, the Netherlands

**Keywords:** Major depressive disorder, Inflammation, Metabolism, Treatment, Randomized controlled trial, Anti-inflammatory medication, Celecoxib

## Abstract

**Introduction:**

As the role of (neuro)inflammation in depression pathophysiology is emerging, augmentation of antidepressant treatments with anti-inflammatory drugs have shown beneficial results, but not consistently across all studies. Inconsistencies may be due to depression biological and clinical heterogeneity. Immuno-Metabolic Depression (IMD) has been put forward as a form of depression characterized by the clustering of low-grade inflammation, metabolic dysregulations and atypical, energy-related symptoms (overeating, weight gain, hypersomnia, fatigue and leaden paralysis). IMD features are present in ∼30% of patients with Major Depressive Disorder (MDD). By selecting these specific patients, directly targeting inflammation may reduce depressive symptoms.

**Methods:**

and analysis INFLAMED is a double-blind randomized controlled trial. 140 MDD patients with IMD characteristics (MDD with Inventory of Depressive Symptomatology (IDS) ≥ 26, IDS atypical, energy related symptoms ≥6, C-Reactive Protein (CRP) > 1 mg/L) will receive either 400 mg celecoxib per day or matching placebo for a period of 12 weeks. Biological, physical and interview data will be collected after 2, 6 and 12 weeks of starting the intervention. Questionnaires will be sent out bi-weekly during the study period. The main study outcome is the IDS (30-item self-report) total score during 12-week follow-up. Secondary study outcomes include response, remission, adverse side effects, symptom profiles (atypical, energy-related symptoms), fatigue, food craving, sleep, anxiety symptoms, functioning, pain, and optionally, microbiome composition. Explorative analyses will be performed on the role of CRP, IL-6, TNF-α, cholesterol, triglycerides, glucose, BMI, waist and hip circumference.

**Ethics and dissemination:**

This protocol has been approved by the Medical Ethics Review Board of the Amsterdam UMC, location VUmc (2022.0015) on 2-6-2022, as well as by the competent authority in The Netherlands: CCMO, on 3-8-2022.

**Registration details:**

Trail registration numbers NCT05415397, EudraCT 2021-003850-21

## Highlights

• This is a large randomized controlled trial to study antidepressant add-on effect of celecoxib in a sample of Major Depressive Disorder (MDD) patients with Immuno-Metabolic Depression (IMD) characteristics.
• This is one of the first studies to specifically look at MDD patients with IMD characteristics.
• This study will examine whether changes in immune-metabolic markers track to changes in depressive symptoms.
• This study is not designed to examine the long-term effects of anti-inflammatory treatment within this group (no data beyond 12 weeks).

## 1 Introduction

Research on the effectiveness of antidepressant treatment has shown that all antidepressants are more effective than a placebo, yet approximately only 50–67% of patients with depression achieve remission from these treatments (Cipriani et al., 2018; Gaynes et al., 2009; Otte et al., 2016). Development of more effective treatments requires a better comprehension and targeting of pathophysiological pathways active in patients showing suboptimal treatment response.

Activation of inflammatory pathways in depression is emerging as a promising target for intervention. Inflammation has been shown to be involved in depression-related pathophysiological mechanisms such as monoaminergic neurotransmission alterations, activation of the indoleamine-pyrrole 2,3-dioxygenase (IDO) enzyme degrading tryptophan towards neurotoxic metabolites, glutamate-related neurotoxicity, and Hypothalamic-Pituitary-Adrenal (HPA)-axis dysregulation (Felger and Lotrich, 2013; Miller and Raison, 2016; Obici et al., 2002; Simmons et al., 2020)

Low-grade inflammation has been linked to poorer treatment response and a more chronic course of depression (Arteaga-Henriquez et al., 2019; Vogelzangs et al., 2014). Multiple meta-analyses in subjects with depression or somatic conditions showed the potential clinical effect of anti-inflammatory medication on depressive symptoms (Kappelmann et al., 2018; Kohler-Forsherg et al., 2019; Wittenberg et al., 2020). For instance, the meta-analyses by Köhler-Forsberg and colleagues (2019) showed, by aggregating a series of four small trials totalling 132 subjects, that antidepressant treatment add-on with the non-steroidal anti-inflammatory drugs (NSAIDs) celecoxib achieved a large effect (d = 0.82) in reducing depressive symptoms compared to placebo. Nevertheless, the statistical heterogeneity in meta-analyses was substantial, suggesting underlying clinical differences in the samples examined. Depression is indeed a heterogeneous disorder, with patients expressing diverse and even opposing symptom profiles potentially linked to different underlying mechanisms, thus requiring different specific treatments (Milaneschi et al., 2020).

Indeed, only a subset of depressed patients show low-grade inflammation (Osimo et al., 2019). Emerging evidence indicates that alterations in inflammatory, metabolic, and bioenergetics biological pathways map more consistently to depressive atypical symptoms, reflecting altered energy intake/expenditure balance (overeating, weight gain, hypersomnia, fatigue, and leaden paralysis). We recently proposed a conceptual model of a depression dimension, labelled immuno-metabolic depression (IMD) (Milaneschi et al., 2020), emerging from the clustering of these biological and clinical characteristics. This dimension will now be applied in the stratification and selection of patients to be matched with targeted anti-inflammatory treatment.

More recently, few clinical trials tried to apply a strategy combining selection of specific patients followed by anti-inflammatory treatment. For instance, the MINDEP study selecting MDD patients with C-Reactive Protein (CRP)>1 mg/l showed a positive effect of minocycline augmentation on depression in post-hoc analyses for patients with elevated inflammation [CRP≥3 mg/l] (Nettis et al., 2021). In contrast, the recently completed PREDDICT RCT aimed to stratify by CRP level in their study comparing NSAID celecoxib or placebo as add-on to vortioxetine. Unfortunately, the study was unable to perform planned analyses and unstratified analyses did not show a positive effect of celecoxib (Baune et al., 2021). Thus with these inconsistent results the efficacy of the potential treatment pathway of anti-inflammatory drugs remains unclear. Recently, more trials have started to target the immune system in depression treatment by selecting specific subset of depressed patients, for instance the SIMCODE trial (Otte et al., 2020) and Insight trial (Khandaker et al., 2018).The INFLAMED trial will add to understanding the antidepressant mechanism of anti-inflammatory drugs, by specifically targeting the inflammatory pathway in patients with IMD characteristics.

The INFLAMED study aims to evaluate add-on efficacy of the anti-inflammatory drug celecoxib [400 mg/day] to currently used evidence-based depression treatments (e.g. selective serotonin reuptake inhibitors (SSRI) or serotonin–norepinephrine reuptake inhibitors (SNRI)), for depressed patients with IMD characteristics. By selecting specific patients, the inflammatory pathway to depressive symptoms will be targeted directly and efficiently. The main hypothesis of the current study is that IMD patients receiving treatment as usual (TAU) + celecoxib, will have lower depressive symptom severity over a 12 week period as compared to IMD patients receiving TAU + placebo. This personalized approach has the potential to lead to large health benefits for a substantial proportion of depressed patients.

### 1.1 Primary objective

The primary objective of this study is to evaluate whether a 12-week celecoxib treatment added to TAU (defined as SSRI or SNRI pharmacotherapy) is more effective in reducing depressive symptoms as measured with the Inventory of Depressive Symptomatology – self report (IDS 30-item self-report (Rush et al., 2006; Rush et al., 1986; Rush et al., 1996; Rush et al., 2011)) than placebo in patients aged 18–65yr with Major Depressive Disorder (MDD) and IMD characteristics (≥6 IDS atypical, energy related symptoms (Lamers et al., 2013) and circulating CRP>1 mg/l) during 12-week follow up.

### 1.2 Secondary objectives

The secondary objective of the study is to examine whether a 12 week celecoxib + TAU is more effective than placebo + TAU in patients aged 18–65 with MDD and IMD characteristics in: reaching treatment response and remission at 12 week follow-up, and improving functioning, lowering fatigue, food craving, and pain at 12 week follow-up.

Additionally, a secondary objective of this study is to better understand how and for whom celecoxib add-on treatment is effective. For this objective, the following biological markers are assessed: CRP, Imterleukine-6 (IL-6), Tumor necrosis factor alpha (TNF-α), cholesterol, triglycerides, glucose, Body Mass Index (BMI), waist and hip circumference. Explorative analyses will be performed on the mediating/moderating role of these markers. These analyses will provide suggestions on whether a potential treatment effect is achieved through modulation of immune-metabolic markers, hypothesized to be at the core of IMD pathophysiology.

## 2 Methods

This protocol was prepared following Standard Protocol Items: Recommendations for Interventional Trials (SPIRIT) guidelines (Chan et al., 2013). The full checklist can be found in Supplement B.

### 2.1 Study design and sample

In total, 140 depressed patients with IMD characteristics (e.g. ≥6 IDS atypical, energy related symptoms and circulating CRP>1 mg/L) and undergoing TAU in primary or secondary care will be randomized (1:1) to celecoxib (400 mg/d) or matched placebo for 12 weeks in a parallel group, superiority framework RCT.

The study will be a monocenter study from the Amsterdam University Medical Centres, location VUmc, department of Psychiatry. Recruitment of participants will be done through a dedicated study website. Local mental health clinics will be approached to refer potential participants to the study website. The study website contains the Patient Information Folder (PIF) and the online screening questionnaire.

### 2.2 Intervention

Patients in this study will receive either an add-on placebo or celecoxib (400 mg/d) in an oral dose of 200 mg per capsule, to be taken twice daily. Celecoxib was chosen as the study medication for four reasons. Firstly, celecoxib is an already available drug since 1999, commonly prescribed for arthritis. Secondly, celecoxib is an affordable generic, with costs less than one euro per capsule. Thirdly, celecoxib has shown most promising preliminary results in depression (Kohler-Forsherg et al., 2019). Finally, celecoxib has a safety profile superior to other NSAIDs (Nurmohamed, 2017).

#### 2.2.1 Choice of dosage

A dosage of 400 mg/day is the oral dosage indicated for the managing of acute pain and previously adopted by all RCT testing the efficacy of celecoxib as add-on to antidepressant treatment in patients with MDD (Kohler-Forsherg et al., 2019; Baune et al., 2021). These studies reported no major adverse events for celecoxib add-on of 400 mg/day. The selected dosage will ensure adequate safety for the study subjects and the possibility to compare results across different studies.

### 2.3 Study procedure

To ensure safe participation and to answer the research questions properly, potential participants will undergo an extensive screener procedure. Full in- and exclusion criteria can be found in Table 1.

**Table 1:** In- and exclusion criteria for the INFLAMED trial.

Inclusion	Exclusion
➢ Age 18–65 years	➢ Contraindications for celecoxib (history of: peptic ulcers, gastrointestinal bleeding, ischemic heart disease, stroke, heart failure, allergic reactions to aspirin/NSAIDs/coxibs; impaired kidney function (creatinine clearance < 30 ml/min); impaired liver function (ALT > 2x upper limit of normal [ULT]))
➢ DSM-5 diagnosis of MDD confirmed with clinical interview (MINI)	➢ Electroconvulsive therapy (ECT) in the past 3 months
➢ Currently using an SSRI or SNRI; subjects should be on the current medication for at least 4 weeks	➢ Being on antidepressants other than SSRIs or SNRIs or being on other psychotropic drugs
➢ IDS score ≥26 and a score ≥6 on atypical, energy-related symptoms scale from IDS ➢ CRP>1 mg/L	➢ Starting other evidence-based non-pharmacological intervention for depression (e.g. psychotherapy) in the 4 weeks before randomization. Clinically overt alcohol/drug dependence or other primary psychiatric diagnoses (schizophrenia, schizoaffective, Obsessive-compulsive disorder (OCD), or bipolar disorder)
➢ A female participant is eligible to participate if one of the following conditions applies: - She is not a woman of child bearing potential (WOCBP) - She is a WOCBP and is not pregnant or breastfeeding. Furthermore, she agrees to use, or is already using, a contraceptive method during the intervention period and up to 1 month after the intervention. A WOCBP must have a negative highly sensitive pregnancy test before the first dose of the study intervention.	➢ Chronic use of anti-inflammatory drugs and corticosteroids ➢ Current use of anticoagulants
➢ Signed informed consent	➢ Not speaking Dutch

### 2.4 Eligibility assessment

Potential participants will enrol through the study website by filling in the IDS and their age as the first steps of screening, after having read the PIF and online consent for screening.

If participants are eligible for the next step (18–65yr, IDS ≥26, atypical, energy related symptoms ≥6), a video-interview will be planned with a trained researcher. In this interview, participants will have the opportunity to ask questions, before signing informed consent to collect data for further screening. After this, participants undergo the interview for the in- and exclusion criteria mentioned in Table 1, including a diagnostic interview to asses for current DSM-5 diagnosis MDD (American Psychiatric Association, 2013).

**Fig. 1:**
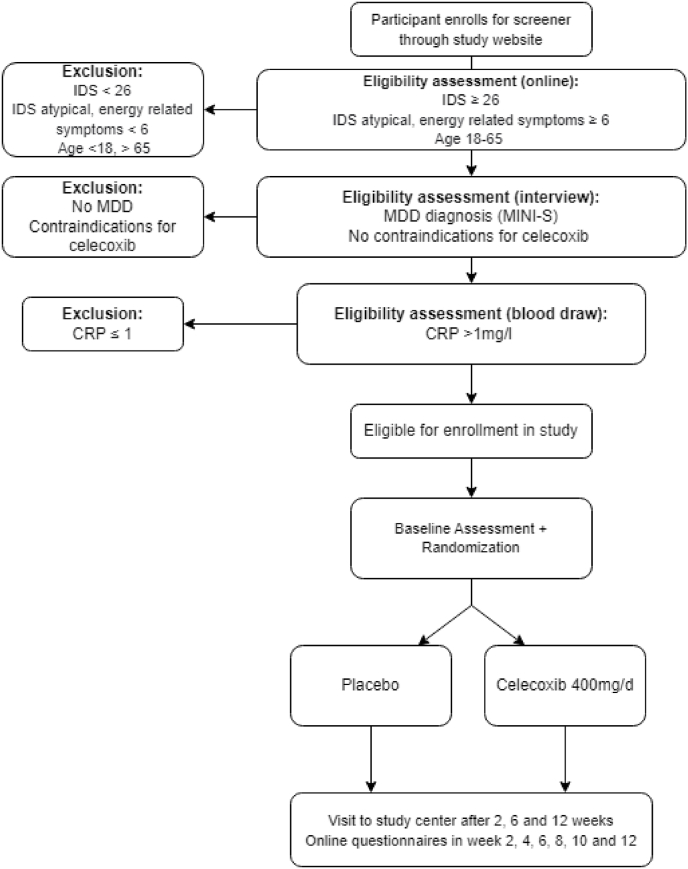
Flow chart of inclusion procedures for the INFLAMED study. IDS: Inventory of Depressive Symptomatology, MDD: Major Depressive Disorder, CRP: C reactive protein.

If still deemed eligible after the interview and after having returned the signed informed consent to researchers, participants will receive a fingerprick-kit for a blood draw at home (or venepuncture at study site if preferred). If a participant has low-grade inflammation (CRP>1) on this test and if there are no signs of liver problems (ALT ≤ 2x upper limit of normal [ULT]) or kidney problems (creatinine clearance ≥30 ml/min), they can be enrolled in the study. The study flow is displayed in Fig. 1.

#### 2.4.1 Concomitant care during the trial

Medication that is contraindicated with celecoxib is not permitted (i.e. anticoagulants). Chronic use of other psychotropic medications, NSAIDS or corticosteroids, are also not permitted. Exceptions are for example made for low dosages psychotropic medications if needed for sleep (for instance, low-dose of mirtazapine, amitriptyline or quetiapine).

Psychotherapy (in any form) is not permitted to start in the 4 weeks before randomization. Participants are permitted to be in therapy for longer than four weeks, or to start a new psychotherapy during study participation. Changes in treatment are recorded during the course of the study.

### 2.5 Baseline data collection

All positively screened participants will be invited for the baseline measure at the study site. Participants have again the opportunity to ask questions about participation, before signing informed consent for study participation. After informed consent is signed, participants are randomized to the celecoxib or placebo group. Participants will then undergo a (sober) blood draw, physical measurements and fill out questionnaires. Finally, at the end of this baseline measurement, participants will receive the study medication for the full duration of the trial from the hospital pharmacy, including clear instructions on usage.

### 2.6 Follow-up measures

Follow-up measures will take place every two weeks after the baseline measures (see Fig. 1), up to the final assessment after twelve weeks. The measures at week 4, 8 and 10 will be online questionnaires. At week 2 and 6 participants are invited to the study site for a blood draw, interview, physical measurements and questionnaires. At these measurements, potential side effects, blood pressure and creatinine levels will be monitored. In case of adverse reaction to the study medication, participants will be pulled out.

The final visit is at week 12. This includes a blood draw, interview, physical measurements and questionnaires, as well as an evaluation of the study by participants, adherence to the protocol, returning any leftover medication and a drug count. Any travel or parking costs made by the participants during the study will be reimbursed. Participants will receive a gift card of €30 for every visit to the research centre.

### 2.7 Outcomes

The main study outcome is the IDS (30-item self-report) total score during 12-week follow-up (Rush et al., 1986, 1996, 2006, 2011). Secondary study outcomes include response (50% reduction in IDS scores), remission (MINI-S diagnostic interview) (Hergueta and Weiler, 2017), adverse side effects for antidepressants (Uher et al., 2009) and for the study medication (as used in the PREDDICT trial (Baune et al., 2021; Fourrier et al., 2018)), symptom profiles (IDS atypical, energy-related symptoms (Lamers et al., 2013);), fatigue (Vercoulen et al., 1994; Beurskens et al., 2000), food craving (Nijs et al., 2007), sleep (Johns, 1991, 1992), anxiety symptoms (Spitzer et al., 2006), functioning (Saltychev et al., 2021), pain (Hawker et al., 2011), and therapy compliance. Additionally, participants have the option to collect a stool sample at baseline and 12 week follow up for microbiome analysis, as well as allow for collection of one additional blood tube for DNA analysis. Baseline measurements will include questionnaires about life events (Brugha and Cragg, 1990), smoking, alcohol use, and physical activity (Craig et al., 2003). A full overview study measures and respective time point can be found in Table 2.

**Table 2:**
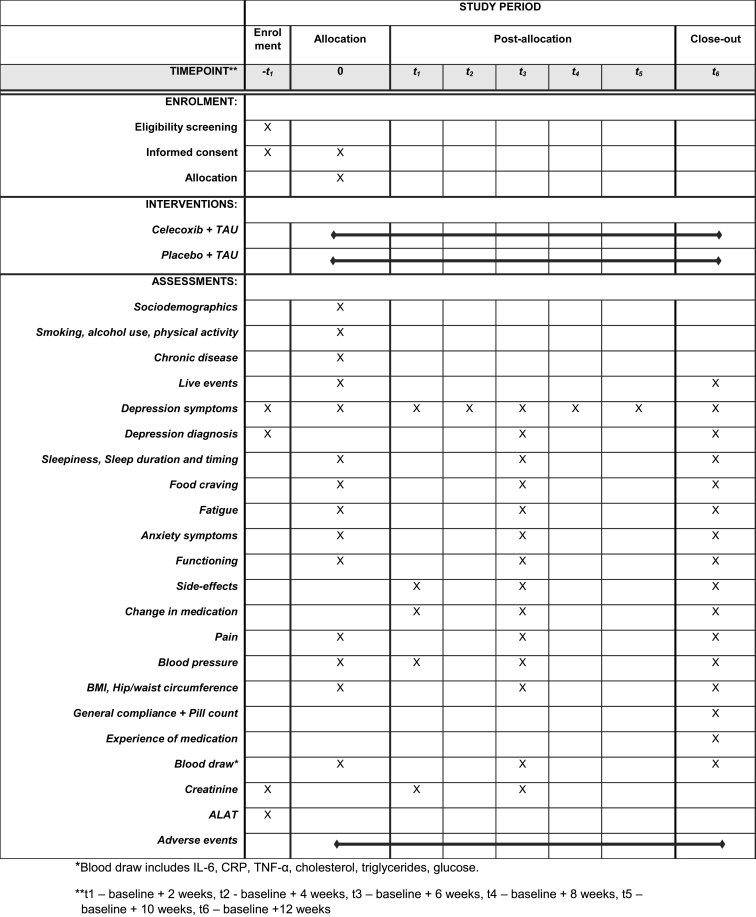
Study measures.

### 2.8 Sample size

In order to properly calibrate the sample size calculation for this longitudinal treatment study we estimated an expected effect size from similar anti-inflammatory add-on published trials. A previous meta-analysis (Köhler‐Forsberg et al., 2019) including 4 RCTs showed a significant effect of celecoxib add-on, as compared to placebo, to standard first line (SSRI/SNRI) antidepressant treatment with a pooled standardized mean difference (SMD) of −0.82 [95%CI: −1.17, −0.46]. We calculated a standardized effect size from the recent PREDDICT trial (Baune et al., 2021) showing no significant difference between placebo versus celecoxib add-on to vortioxetine. From this trial, the differences in means in the two treatment groups at the end of the treatment were standardized using baseline standard deviations, obtaining a SMD of 0.30 [95%CI: −0.06, 0.66]. We pooled the effect sizes for all the 5 trials mentioned above using a random effect meta-analysis (see forest plot below). The pooled SMD was −0.52 [95%CI: −1.05, 0.01] which we used in subsequent power calculations.

**Fig. 2:**
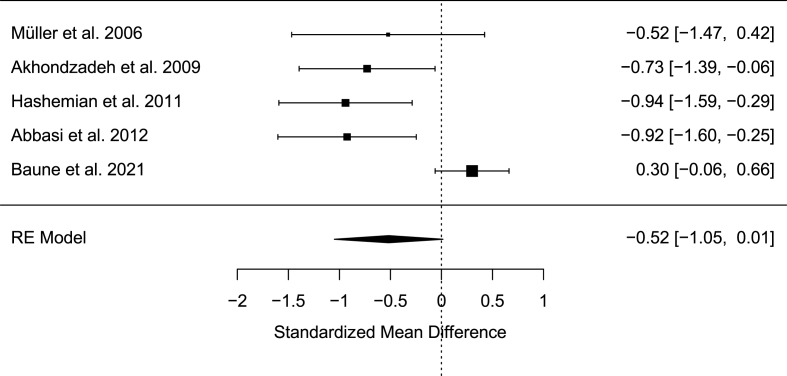
Standardized Mean Difference of previous RCTs.

Power calculations were performed with the “powerlmm” package (Power Calculations for Longitudinal Multilevel Models, v. 0.4.0) in R v4.0.0 (R Project for Statistical Computing), which is based on the analytical model described in Galbraith and Marschner (2002). We considered a study design with 6 follow-up repeated assessments of depressive symptoms with an ICC of 0.5 and an overall SMD of −0.52 (see Fig. 2) between treatment groups (1:1 ratio) across the follow-ups. We additionally modelled attrition based on the recent results of the PREDDICT trial (Baune et al., 2021), reporting drop-outs of 16% in the placebo add-on group and 12% in the celecoxib add-on at the end of the treatment. Under these assumptions, a sample size of 140 subjects allows to detect a significant (α = 0.05) treatment-by-time interaction effect with 83% statistical power.

### 2.9 Randomization

Double-blind randomization is performed using block randomization in Castor EDC (ratio 1:1, celecoxib n = 70, placebo n = 70). The randomization codes are kept by the hospital pharmacy, no involved researchers have access to the randomization list. Both capsules and bottles of the placebo and study medication are identical in appearance. For safety reasons participants can be deblinded by a designated staff member, not involved with the study, if deemed necessary.

### 2.10 Data analysis

Analyses will be performed following an Intention-To-Treat principle: all participants will be included in the analysis according to their allocated treatment group at randomization. The primary outcome will be analysed using data from all post-randomization time points: 2-week, 4-week, 6-week, 8-week, 10-week and 12-week assessments. Given the correlation between repeated measures within subjects, linear mixed models will be used including a random intercept at subject level. Linear mixed models are able to handle missing data using maximum likelihood estimation. In the primary analysis, fixed effects will consist of treatment, a linear effect of time, and a treatment-by-time interaction. In secondary analyses, time will be dummy-coded and all two-way interactions between treatment and time dummies will be entered in the model to allow the estimation of group means at each time point. Estimates will be adjusted for the baseline IDS scores. Standardized effect sizes will be computed by dividing the estimated mean treatment group difference (across the overall follow-up and at each follow-up assessment) by the SD of the baseline outcome. The statistical significance threshold will be set at α = 0.05, two-sided. Analysis for the secondary outcomes will follow the same approach as the primary outcomes. Descriptive statistics will be calculated for adverse events. All statistical analyses will be performed using R or SPSS.

### 2.11 Data management

All potential study participants will be assigned an screener ID number. If eligible for participation, a new, unique five digit ID will be assigned to the participant. All data will be pseudonymised and stored in a secure, password-protected database, to which only study members have access to. Data will be entered into the electronic case report forms (eCRF – Castor EDC). This eCRF ensures a safe remote server for storage and daily backups. After completing the trial, the database will be locked and data transferred to the protected server of the Amsterdam UMC. Only assigned and trained team members will have access to the data and the key that connects ID number to personal data.

Bodily material (e.g. blood, stool samples) will be coded and stored for a maximum of 25 years after completion of the study in the Amsterdam UMC Biobank. These samples may be used for additional research. Participants give explicit consent for storage and may invoke consent for storage at any time.

### 2.12 Monitoring

A monitor plan has been realised and monitor visits are planned. Considering the low risk profile of the study and the absence of an interim analysis, no Data Safety Monitoring Board has been installed. (Serious) Adverse Events and Suspected Unexpected Serious Adverse Events will be reported to the Medical Ethics Review Board. Additionally a yearly safety report will be sent to the Medical Ethics Review Board.

## Ethics and dissemination

This study will be conducted according to the principles of the Declaration of Helsinki (version 9th July 2013) and in accordance with the Medical Research Involving Human Subjects Act (WMO), the EU Clinical Trial Directive (Directive, 2001/20/EC) and the subsequent Clinical Trials Regulation (EU) No 536/2014 and ICH GCP E6(R2) Guideline.

### Consent

Separate informed consents will be obtained. As described in the methods, participants read the PIF before giving consent for the online questionnaire (consent 1a). After this, participants receive a PIF at home and provide written consent for the screener interview and blood draw () and again a written consent for participation in the study (consent 2). By signing consent 2, participants agree to the GP being contacted by the research staff to inform on participation and to report any clinical outcomes deemed necessary by the research staff. Additionally, participants are asked to sign consent for Biobanking. This includes the storage of collected materials up to 25 years, with an optional consent to use this material for research outside the scope of the current protocol. Participants may withdraw consent at any point in time, without providing a reason.

### Ethics approval

This protocol has been approved by the Medical Ethics Review Board of the Amsterdam UMC, location VUmc (2022.0015) on 2-6-2022, as well as by the competent authority in The Netherlands: CCMO, on 3-8-2022.

### Dissemination plan

Results of this trial will be published in peer-reviewed international journals. Results and implications will also be communicated with patients through the Brain Foundation Netherlands, Depressie Vereniging and through the study website (www.immunometaboledepressie.nl). Additionally, findings will be communicated to appropriate national and international media for a lay audience.

## Administrative information

Protocol version 1.6–02-11-2022.

## Funding

The INFLAMED trial is funded with a grant from the 10.13039/501100000942Brain Foundation Netherlands (Hersenstichting), grant number DR-2020-00367. The study is sponsored by Amsterdam UMC, Vrije Universiteit, Amsterdam, the Netherlands.

## Registration details

Trail registration numbers NCT05415397, EudraCT 2021-003850-21.

## Contributors

YM and FL designed the study, obtained funding, developed protocol and study materials, obtained necessary approvals for the study and revised this manuscript. JCZ contributed to study design, developed protocol and study materials, obtained necessary approvals for the study and wrote the first draft for this manuscript. CHV and BWJHP contributed to study design, obtaining funding, development of the protocol and revised this manuscript. PMB, DR and MTH contributed to study design, protocol and revised this manuscript. CHV, BWJHP, PMB. MTH, DR, JCZ are part of the study management group. FL, YM and CHV are the principal investigators, lead researchers and guarantor of the study.

## Declaration of competing interest

The authors report no conflicts of interests.
